# Copper metabolism in osteoarthritis and its relation to oxidative stress and ferroptosis in chondrocytes

**DOI:** 10.3389/fmolb.2024.1472492

**Published:** 2024-09-11

**Authors:** Qingyuan Yu, Yanan Xiao, Mengqi Guan, Xianshuai Zhang, Jianan Yu, Mingze Han, Zhenhua Li

**Affiliations:** ^1^ Clinical College of Integrated Traditional Chinese and Western Medicine, Changchun University of Traditional Chinese Medicine, Changchun, China; ^2^ Orthopedic Center, Affiliated Hospital of Changchun University of Traditional Chinese Medicine, Changchun, China

**Keywords:** ferroptosis, copper homeostasis, oxidative stress, osteoarthritis, chondrocytes, dual regulation, cuproptosis ferroptosis, cuproptosis

## Abstract

Ferroptosis, an iron-ion-dependent process of lipid peroxidation, damages the plasma membrane, leading to non-programmed cell death. Osteoarthritis (OA), a prevalent chronic degenerative joint disease among middle-aged and older adults, is characterized by chondrocyte damage or loss. Emerging evidence indicates that chondrocyte ferroptosis plays a role in OA development. However, most research has concentrated on ferroptosis regulation involving typical iron ions, potentially neglecting the significance of elevated copper ions in both serum and joint fluid of patients with OA. This review aims to fill this gap by systematically examining the interplay between copper metabolism, oxidative stress, ferroptosis, and copper-associated cell death in OA. It will provide a comprehensive overview of copper ions’ role in regulating ferroptosis and their dual role in OA. This approach seeks to offer new insights for further research, prevention, and treatment of OA.

## 1 Introduction

Osteoarthritis (OA), a prevalent chronic degenerative bone and joint disease among middle-aged and elderly individuals, is a leading cause of disability in the elderly (Hunter and Bierma-Zeinstra, 2019). The condition most commonly affects the knee and is more prevalent in older women than men, potentially linked to estrogen levels (Hunter and Bierma-Zeinstra, 2019). OA significantly impairs mobility and quality of life, leading to anatomical remodeling of bones and joints (Bland and Cooper, 1984). The primary symptom of OA is pain, accompanied by joint stiffness, movement restriction, and dysfunction, which ultimately results in complete joint damage. Pathological changes in OA include extracellular matrix degradation, osteoarticular cartilage loss, subchondral bone ossification, synovitis, bony growths, and reduced function of surrounding muscles and ligaments (Martel-Pelletier et al., 2016). Risk factors for OA are diverse and include genetic predisposition (40%–65% of the risk, with hands and hips being more genetically influenced than knees) (Rahman et al., 2014), joint morphology, body mass index, bone mineral density (Hochberg et al., 2013), age (Helmick et al., 2008), gender (Hunter and Bierma-Zeinstra, 2019), congenital anatomical factors (Sharma et al., 2013), hyperlipidemia (Driban et al., 2016), synovitis (Tu et al., 2019), and functional factors such as dysfunctional muscle contraction and high-intensity exercise (Wang et al., 2012). Age remains the strongest risk factor (Helmick et al., 2008), contributing to OA through cellular aging processes, SASP factor production, and immune-related factors (Diekman et al., 2018). OA arises from multiple imbalances, including oxidant-antioxidant imbalance, anabolic-catabolic metabolism imbalance, osteoblast-osteoclast imbalance (Zhou et al., 2020), and cartilage homeostasis disruption. Research increasingly indicates that OA development is multifactorial, involving injury, metabolic disorders, mechanical stress, inflammatory aging, oxidative stress, mitochondrial damage, and immune cell involvement (Jeon et al., 2018).

Abnormal copper content has been observed in patients with OA (Yang et al., 2023), suggesting that imbalances in copper metabolism and homeostasis may contribute to OA prevalence through various biological effects. We categorize copper metabolism into four states: normal physiological levels, beneficial copper ions, copper deficiency, and copper overload. This review explores these states in relation to bone metabolism pathways and their impact on chondrocyte ferroptosis sensitivity. Insights from this review may inform future research on OA etiology, mechanisms, and treatment strategies (Table 1).

**TABLE 1:** The regulatory function exhibited by varying concentrations of copper.

Markers		Copper ion concentration		
		Copper homeostasis	Copper deficiency	Copper overload
bone metabolism	bone anabolism	ALP↑、IGFBP-3↑、COL1A1↑、OCN↑、 OPN↑、COL-2↑、ACAN↑、LOX↑ (Han J. et al., 2024a; Ciaffaglione and Rizzarelli, 2023; Lin et al., 2019; Liu et al., 2022; Wang et al., 2023a)	MAO↓、LOX↓、 ALP↑、COL1A1↑、OPG↑、OPN↑ (Han J. et al., 2024a; Ciaffaglione and Rizzarelli, 2023)	TGF-β↑、ALP↓、COL1A1↓OCN↓ 、 IGF-1↓ 、BMP-2 ↓ (Cui et al., 2022; Ciosek et al., 2023)
	bone catabolism	MMP9↑、TIMP-3↑、 MMP13↓MMP1↑ (Wang et al., 2023a; Hughes et al., 2008; Zhang et al., 2024)	MMP9 ↑、MMP2 ↑ (Lentsch et al., 2001)	MMP3↑、MMP13↑、 ADAMTSs↑ (Lin et al., 2019)
inflammatory factors		IL-18↓、IL-10↑、1L-1↑、1L-6 ↑、1L −8↑、TNF-α ↓ (Ciaffaglione and Rizzarelli, 2023; Lin et al., 2019; Wu et al., 2023a; Liu et al., 2023)	1L-8 ↓、 1L-6 ↓、1L-1α (Chen et al., 2022)↓	1L-1β↑、1L-18 ↑、 1L-6 ↑、1L −8↑ (Lin et al., 2019; Zhao et al., 2022a)
immune factors		IL-12↑、TGF-β↓、 IL-10↓ (Cheng et al., 2022)	IL-2↓、PD-L1↓ (Cheng et al., 2022; Voli et al., 2020)	PD-L1↑ (Voli et al., 2020)
ferroptosis		CP ↑、SLC7A11↑ GPX4↑ (Xue et al., 2023b)	SOD1↓、SLC7A11↓、 CP↓ (Liu et al., 2023; Xue et al., 2023a; Jiayi et al., 2024)	FTH-1↓、MTF1↑、FPN ↓、GPX4↓、GSH↓、HAMP↑、 HO-1↑、GLS2↑ (Liu et al., 2023; Jiayi et al., 2024)
cuproptosis				MTF1 ↑ 、CTR1↑、GPX4↓、GSH↓、MTs↑、ATP7A ↓ (Zhao et al., 2022b; Jiayi et al., 2024; Xie et al., 2023; Li et al., 2023)
oxidative stress		COX↑、 LOX ↑、 SOD1↑ (Vo et al., 2024)	SOD↓、CCO↓、 GPx ↓、NOX↑、CP↓、CAT↓、GPX4、SOD1↓ (Jiayi et al., 2024; Wang et al., 2023b)	NQO1↑、GSH↓、 P62↓、NFR2↓ (Jiayi et al., 2024)

## 2 Copper metabolism

Copper (Cu), a critical element for biological functions, serves as a cofactor for several essential enzymes, including lysyl oxidase (LOX) (Vallet and Ricard-Blum, 2019), superoxide dismutase (SOD) (Trist et al., 2021; Sun et al., 2024), and cytochrome oxidase (Swaminathan and Gohil, 2022). Beyond its role in enzyme catalysis, copper interacts with iron metabolism by influencing electron transport chain enzymes, iron-containing enzymes, and iron storage proteins such as ferritins, ceruloplasmin (Cheng and Li, 2007). Ceruloplasmin (CP), a key protein involved in copper transport and iron oxidase activity, plays a crucial role in maintaining and regulating copper and iron homeostasis, serving as a pivotal intersection between copper and iron metabolism (Skarżyńska et al., 2024). Copper regulates kinases such as MEK, ULK1, CK2, and PDK, thereby affecting cell proliferation and differentiation through modulation of downstream signaling factors (Tsang et al., 2020; Guo et al., 2021; Chojnowski et al., 2022; Sun et al., 2024). It also activates various transcription factors involved in mitochondrial respiration, antioxidant defense, lipolysis, and ferro-oxidase activity related to iron absorption, osteogenesis, osteoclast differentiation, and bone metabolic homeostasis. Proper copper homeostasis is essential for maintaining muscle and bone health (Dollwet and Sorenson, 1988) and for managing bone and joint disorders (Liu et al., 2022). This review will detail the maintenance of copper homeostasis under physiological conditions, examine copper’s role in bone and osteoarthritic cell metabolism, and explore the impact of copper imbalance on bone homeostasis while addressing current research gaps and offering constructive perspectives.

Copper exerts a biphasic regulatory influence on immunomodulation and the inflammatory response, contingent upon its dosage. Empirical evidence indicates that optimal concentrations of copper and copper-containing compounds can downregulate pro-inflammatory cytokines, including IL-6 and TNF-α, while up-regulating the anti-inflammatory cytokine IL-10. This modulation results in the suppression of inflammatory responses in macrophages and chondrocytes, thereby manifesting an anti-inflammatory effect (Xu C. et al., 2018; Hou et al., 2019; Lin et al., 2019). However, when the copper dosage surpassed a specific threshold, the resulting excess copper induced the upregulation of inflammatory mediators, including IL-6, IL-1β, IL-8, TNF-α, iNOS, and COX-2, thereby promoting pro-inflammatory effects (Pereira et al., 2016). Chronic inflammation is a hallmark of cartilage degradation in osteoarthritis (OA), and thus, reducing copper concentrations in OA-affected joints may represent a significant strategy for anti-inflammatory intervention and attenuation of OA-related damage.Furthermore, optimal concentrations of copper ions facilitate the polarization of macrophages towards an anti-inflammatory phenotype, thereby mitigating the progression of synovial inflammation during remission (Xu X. et al., 2018; Lin et al., 2019). Empirical evidence indicates that macrophages in a high-copper milieu suppress the immune response through the secretion of TGF-β (Lee et al., 2018)and IL-10 (Díez-Tercero et al., 2021). Conversely, a low-copper environment similarly exerts an immunosuppressive effect. This is evidenced by a reduction in antibody production by B cells, decreased IL-2 secretion by T cells, and a reduction in neutrophil counts, collectively leading to compromised immune function (Cheng et al., 2022).

### 2.1 Copper metabolism in physiological states (crosstalk with iron metabolism)

#### 2.1.1 Absorption and distribution of Cu

**FIGURE 1:**
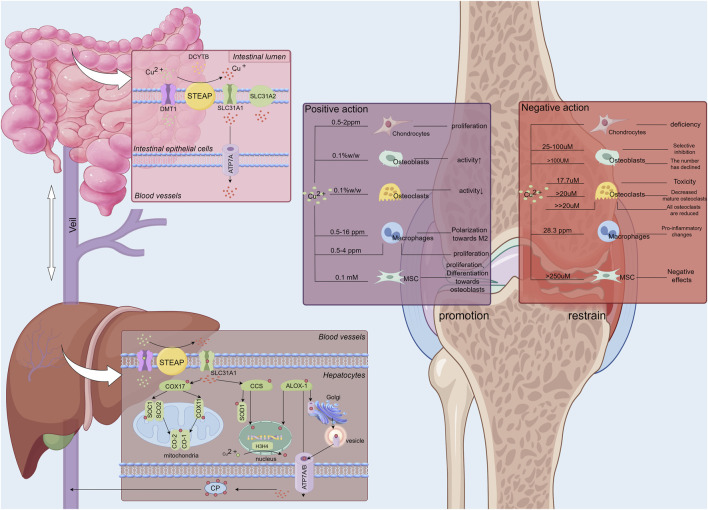
The absorption, transportation, and storage processes of copper ions within the human body, alongside the regulatory effects of varying copper ion concentrations on bone and chondrocytes. Copper ions (Cu^2+^) ingested by the human body are absorbed through the intestinal lumen, where DCYTB and small intestinal epithelial cells reduce Cu^2+^ to Cu^+^. Cu^+^ is then absorbed into the small intestinal epithelial cells by SLC31A11 and secreted extracellularly into the bloodstream via ATP7A. Once in the bloodstream, copper ions travel to the liver via the portal vein. In the liver, copper ions are absorbed by hepatocytes, regulating their activity. The liver, serving as a major storage site for copper ions, releases them into the blood, where they are distributed to various tissues and organs, including bones and joints, to exert their biological effects via carriers like ceruloplasmin.Copper ions exhibit a dual regulatory effect on bone and articular cartilage depending on their concentration. At a Cu^2+^ concentration of 17.7 µM, toxic effects on osteoclasts are observed (Schamel et al., 2017). A significant reduction in the number of mature osteoclasts occurs when Cu^2+^ concentrations exceed 20 µM (Bernhardt et al., 2021). At concentrations substantially greater than 20 μM, a marked decrease in the number of osteoclasts at all developmental stages is noted (Bernhardt et al., 2021). For osteoblasts, Cu^2+^ concentrations ranging from 25 to 100 µM selectively inhibit osteoblastic activity. Structural damage to osteoblasts occurs at concentrations exceeding 100 µM (León et al., 2014). In contrast, mesenchymal stem cells exhibit greater tolerance to elevated copper levels compared to osteoblasts and osteoclasts, experiencing detrimental effects only at Cu^2+^ concentrations exceeding 250 µM. The primary impact on mesenchymal stem cells at these higher concentrations is regulatory rather than destructive (Schamel et al., 2017).

The absorption, transport, and excretion of copper (Cu) within the body are both intuitive and complex (Liao et al., 2022) (Figure 1). Dietary Cu^2+^ is absorbed as Cu^+^ by intestinal epithelial cells through SLC31A1 (CTR1), facilitated by STEAP and DCYTB reduction, or via vesicle-associated SLC31A2 (CTR2), which differ in their Cu^+^ affinities (Tsai et al., 2015). Once inside the cell, Cu^+^ binds to chaperones such as COX17, CCS, and ATOX1, and is transported to various organelles, including the Golgi, mitochondria, and nucleus, where it performs critical functions (McKie et al., 2001; Ohgami et al., 2006; Chen et al., 2020). Cu^+^ transported to SOD1 in the cytoplasm and inner mitochondrial membrane via CCS helps regulate SOD1 balance, reducing excess ROS produced by mitochondria and mitigating oxidative stress (Wong et al., 2000). In mitochondria, Cu^+^ is transported from the cytoplasm to SCO1 cysteine residues by COX17, forming disulfide bonds and integrating into MT-CO2/COX2 (Pierson et al., 2019). COX17 also aids in the oxidative respiratory chain by transferring Cu^+^ to MT-CO1/COX1 and COX11 (Cobine et al., 2006). Additionally, ATOX1 transports Cu^+^ to the Golgi, where it binds to ATP7A/B (Hamza et al., 2001), facilitating intracellular Cu^+^ excretion and regulating elevated Cu^+^ levels through Golgi-lysosomal translocation (Horn and Wittung-Stafshede, 2021). Glutathione (GSH) stabilizes ATP7A/B by maintaining redox reactions (Chen et al., 2020). Furthermore, ATOX1, along with CCS, transports Cu^+^ to the nucleus (Beaino et al., 2014), activating HIF-1 (Feng et al., 2009) and influencing transcription (Beaino et al., 2014). Recent studies suggest that MEMO1 enhances ATOX1’s binding affinity for Cu^+^, reducing ROS oxidative stress via a Fenton-like reaction (Zhang et al., 2022), and ATOX1 serves as a target for mitigating intracellular free Cu^+^ damage. Additionally, prion protein (PRNP), a widely distributed glycoprotein, is essential for copper homeostasis regulation and may be involved in ROS-associated ferroptosis (Lin C. et al., 2023), which will be explored further below.

#### 2.1.2 The processes of copper excretion and transport, along with the regulation of iron, are critical physiological functions

##### 2.1.2.1 ATP7A, ATP7B

ATP7A and ATP7B are essential for intracellular copper (Cu) regulation and secretion (Dmitriev and Patry, 2024). ATP7A and ATP7B play a critical role in maintaining copper homeostasis *in vivo*. Both proteins are localized within the intracellular Golgi network and shuttle between the Golgi apparatus and the plasma membrane (Lutsenko et al., 2007; Kaler, 2011; Polishchuk and Lutsenko, 2013). Nevertheless, their functions exhibit distinct differences.ATP7A is predominantly localized to the apical membrane of polarized cells, where it plays a crucial role in facilitating copper absorption in the intestine and its subsequent transport into the bloodstream (Bartee and Lutsenko, 2007). In contrast, ATP7B is primarily situated on the basolateral membrane, where it is involved in the process of hepatic copper excretion (Bartee and Lutsenko, 2007). When either ATP7A or ATP7B is dysfunctional, dysregulation of copper metabolism ensues. Specifically, mutations in ATP7A lead to Menkes disease, while mutations in ATP7B result in Wilson’s disease (Fieten et al., 2016; Chen et al., 2020). Ceruloplasmin (CP) requires the binding of six copper ions to function as an iron-oxidizing enzyme. The functionality of ATP7B, which facilitates copper transport to CP, is therefore crucial for the coordination of copper metabolism with iron metabolism (Wittung-Stafshede, 2015).Upon cellular entry, copper (Cu) is mediated by the CysXXCys motif of ATOX1 (Mangini et al., 2022; Yang F. et al., 2022; Weishaupt et al., 2024), facilitating its translocation to the metal-binding domains of ATP7A and ATP7B within the Golgi apparatus. This process enables the incorporation of Cu into the trans-Golgi network via the binding actions of ATP7A and ATP7B (Ruturaj et al., 2024). Subsequently, Cu functions as a cofactor, contributing to the regulation of the activity and composition of various copper-dependent enzymes, such as lysyl oxidase (LOX), within the Golgi (Ruturaj et al., 2024; Whitlow et al., 2023). When intracellular Cu levels rise, ATP7B mediate its excretion through the Golgi-vesicle-plasma membrane pathway (Petris et al., 1996; Schaefer et al., 1999), ATP7A predominantly facilitates the metalation of copper-dependent enzymes within the Golgi apparatus (Chen et al., 2020), a key mechanism for maintaining copper homeostasis. The interaction between ATP7A, ATP7B and AP1/2 ensures the precise localization and efficient transport of Cu (Hirst et al., 2012; Dmitriev and Patry, 2024; Ruturaj et al., 2024). Furthermore, optimal copper levels enhance ATP7A abundance (Whitlow et al., 2023), and in hepatocytes, ATP7B is essential for the metalation of ceruloplasmin, thus enhancing CP activity (Terada et al., 1999; Yanagimoto et al., 2009). The regulation of copper enzyme composition and activity by ATP7A and ATP7B, as well as the excretion of excess Cu, is vital for maintaining cellular copper homeostasis and redox balance (Dmitriev and Patry, 2024). Research indicates that during intervertebral disc degeneration (IDD) (Gu et al., 2024), oxidative stress exacerbates copper overload-induced damage, promoting copper-dependent cell death through upregulation of SP1/CRT1 and FDX1 (Chen J. et al., 2024). ATP7A and ATP7B has been observed to mitigate this pathological process (Chen K. et al., 2024).

Dysfunction or aberrant degradation of ATP7A and ATP7B, caused by mutations, gene knockout (Gao et al., 2021; Kabin et al., 2023), drug effects (Gao et al., 2021), or pathological metabolism, disrupts intracellular copper homeostasis and impairs copper-dependent enzymes (Baker et al., 2013), leading to cellular damage and disease progression (Fodor et al., 2023). Elesclomol, a Cu^2+^ carrier, induces ATP7A degradation through direct interaction (Kirshner et al., 2008), resulting in abnormal mitochondrial Cu accumulation, significant ROS production, and SLC7A11 degradation (Gao et al., 2021), culminating in copper-dependent ferroptosis (Gao et al., 2021). This underscores the interplay between copper and iron metabolism, chondrocyte ferroptosis, and copper-induced cell death. Previous studies have shown that Cu can modulate Fe levels by upregulating mRNA levels of *CTR1, FPN, and TFR* in iron-deficient intestines (Evcan and Gulec, 2022). This regulation is attributed to Cu’s impact on HIF-2α stability (Tan et al., 2021), which affects CTR1 and FPN expression (Anderson et al., 2013). The balance between ATP7A,ATP7B, CTR1, and FPN1 may serve as a critical link between Cu and Fe metabolism, ferroptosis, and cuproptosis. This balance presents a potential target for modulating ferroptosis-related injury through copper metabolism. The subsequent section will offer a detailed discussion on the interconnections between Cu and Fe death, providing valuable insights into this regulatory mechanism.

##### 2.1.2.2 Ceruloplasmin

Ceruloplasmin (CP) plays a critical role in Cu metabolism by receiving Cu^+^ ions secreted into the bloodstream, oxidizing them to Cu^2+^, and transporting approximately 95% of these ions to support physiological functions. CP is integral to the regulation of both Cu and Fe transport, and its redox activity is essential for maintaining Cu/Fe homeostasis (Skarżyńska et al., 2024). Physiological Cu concentrations are vital for sustaining the activity of CP oxidases, including amine oxidases, which are critical for preserving CP function (Yiannikourides and Latunde-Dada, 2019). CP facilitates the oxidation of ferrous iron (Fe^2+^) exported by ferroportin (FPN) to ferric iron (Fe^3+^) for transport via transferrin (TF) in the bloodstream (Anderson and Vulpe, 2009). Additionally, CP interacts with iron metabolism-related proteins such as transferrin (TF) and lactoferrin (LF) (Van Eden and Aust, 2000; Ha-Duong et al., 2010), playing a pivotal role in regulating iron export processes and maintaining iron homeostasis (Skarżyńska et al., 2024). CP also binds lactoferrin (LF) and inhibits myeloperoxidase (MPO) (Chapman et al., 2013), exerting anti-inflammatory and antioxidative stress effects (Kolstø Otnaess et al., 1983; Mayyas et al., 2014; Rocha-Penha et al., 2017; Cecchini Gualandi and Boni, 2023; Elizarova et al., 2023). A deficiency in CP leads to iron overload in most tissues and cells (Xu et al., 2004; Zheng et al., 2018; Villadsen et al., 2023). Regarding lipid metabolism, CP-deficient mice show increased blood triglyceride levels and disrupted lipid metabolism in hepatic and adipose tissues. Exogenous CP administration ameliorates these disturbances and upregulates the expression of iron-associated proteins, such as TFR1 and ferritin (Raia et al., 2023).In summary, CP mediates the regulation of copper (Cu) and iron (Fe) metabolism, redox homeostasis, and lipid metabolism. The interplay between Cu and Fe is critical, as Cu facilitates iron uptake, excretion, transport, and mitochondrial utilization (Doguer et al., 2018; Ruiz et al., 2021), while iron can inhibit Cu (Doguer et al., 2018). This negative feedback mechanism is essential for coordinating physiological Cu/Fe metabolism. Research indicates that the progression of OA (Kolhe et al., 2020) and rheumatoid arthritis (RA) (Shan et al., 2019) is associated with elevated levels of Cu and CP (Bhattacharya et al., 2012). While it is hypothesized that increased CP may contribute to OA pathogenesis, these mechanisms are not yet fully understood (Babinets et al., 2017). Given CP’s role in copper and iron metabolism, lipid metabolism, and redox reactions, changes in copper levels could influence CP’s regulatory functions related to ferroptosis (Shang et al., 2024a) and oxidative stress (Skrzep-Poloczek et al., 2020). This topic will be further explored in the subsequent discussion.

### 2.2 Positive protective effect of copper on bone and cartilage

The human body contains between 50 and 120 mg of copper (Cu), with approximately 70% of it localized in muscle and bone tissues (Olivares and Uauy, 1996; Turnlund et al., 1998). In bone tissue, the physiological concentration of Cu is typically 5–6 mg/kg dry weight. Copper is essential for regulating collagen production, bone and cartilage matrix metabolism, and remodeling processes. It also enhances bone density and strength, promoting normal bone development and maintaining bone and cartilage homeostasis (Wang et al., 2016; Baino et al., 2018; Antoniadis et al., 2019; Ciosek et al., 2023). This paper will explore Cu’s regulatory effects on various cellular components within bone and joint tissues.

#### 2.2.1 Chondrocyte

Research has shown that copper content in cartilage is significantly higher than in other tissues under physiological conditions (Roczniak et al., 2017), highlighting its key role in cartilage metabolism. One of the main challenges in treating OA is the presence of defective chondrocytes (Wang et al., 2021), emphasizing the need to investigate the relationship between copper and cartilage. Physiological levels of copper are essential for maintaining normal chondrocyte metabolism through various mechanisms. Copper facilitates chondrocyte proliferation and cartilage repair by contributing to the secretion of chondrogenic regulatory proteins, including insulin-like growth factor 1 (IGF-1), insulin-like growth factor-binding protein 3 (IGFBP-3), and alkaline phosphatase (Makris et al., 2013). Optimal copper concentrations also enhance the differentiation of bone marrow-derived mesenchymal stem cells (BMSCs) into chondrocytes (Xu C. et al., 2018).Copper functions as a vital cofactor for lysyl oxidase (LOX), facilitating collagen cross-linking within cartilage by enhancing LOX activity and the activity of collagen cross-linking enzymes (Makris et al., 2013; Lin et al., 2020). Additionally, copper regulates the conformational stability of hypoxia-inducible factor 1 (HIF-1) (Amarilio et al., 2007) and mediates the expression of key extracellular matrix (ECM) components, such as aggrecan (Acan) and collagen type II alpha 1 (Col2A1), through the transcription factor SOX9. Copper also counteracts the upregulation of markers associated with bone catabolism, which is essential for maintaining cartilage integrity (Gee et al., 2007; Lu et al., 2022).

Building on the previously discussed beneficial effects of copper ions on cartilage metabolism, research has established a significant link between copper and OA treatment. Empirical evidence suggests that Cu^2+^ combined with HyCar can elicit effects comparable to those of superoxide dismutase (SOD). Additionally, copper facilitates the nuclear translocation of nuclear factor erythroid 2-related factor 2 (NRF2) and activates the NRF2/heme oxygenase-1 (HO-1) pathway, mitigating oxidative stress and inflammation through interactions with hyaluronic acid (Hy) and carnosine (Car) (Bellia et al., 2023). An increasing body of research is focusing on copper and its derivatives for OA treatment, underscoring copper’s significant potential as a metal-coordinated therapeutic agent in managing OA.

Metal-organic frameworks (MOFs) incorporating copper and active compounds, such as CuMH and B2M-CuS nanoparticles (Wang H. et al., 2023), exhibit considerable potential for treating OA (Cao et al., 2023). For example, copper can form MOFs with therapeutic agents like MH (Caselli et al., 2016), demonstrating notable efficacy in OA management. Specifically, intra-articular injection of CuMH has been shown to reduce reactive oxygen species (ROS) levels in patients with OA and chondrocytes exposed to hydrogen peroxide. This treatment stabilizes mitochondrial function, enhances superoxide dismutase (SOD) activity, and downregulates interleukin-6 (IL-6). Additionally, CuMH decreases the expression levels of inflammatory and catabolic markers, such as matrix metalloproteinases (MMPs), while up-regulating anabolic markers related to cartilage, such as collagen type II alpha 1 (Col2A1) (Cai et al., 2023).Recent research has highlighted a copper-based metal-organic framework (Cu MOF) nanomase as a significant factor in OA pathogenesis. Cu MOF nanomase has been shown to lower intracellular ROS levels, inhibit cartilage matrix degradation, and promote anti-inflammatory macrophage polarization (Yu et al., 2024). The importance of copper-related MOFs (Cu-MOFs) lies in their ability to synergistically combine optimal concentrations of copper ions with therapeutic effects for OA, enhancing the inhibition of OA progression more effectively than drug administration alone. Further exploration and development of Cu-MOFs could significantly advance OA treatment, expanding the therapeutic potential of copper in managing this condition.

#### 2.2.2 Mesenchymal stem cells, osteoblasts and osteoclasts

From an osteogenic perspective, copper (Cu) plays a pivotal role in enhancing bone formation by upregulating key bone-related genes, including alkaline phosphatase (ALP), osteopontin (OPN), osteocalcin (OCN), collagen type I (Col I), and vascular endothelial growth factor (VEGF) in endothelial cells. Copper stimulates the proliferation and activity of osteoblasts (OBs) (Wang J. et al., 2024), aiding in bone mineralization, essential for bone development and remodeling (Pucheu et al., 1995; Li et al., 2021). Furthermore, elevated VEGF levels induced by copper enhance the vascularization of bone tissue by promoting the formation (Wu et al., 2013), migration, and permeability of endothelial cells (Zong et al., 2017). This vascularization is critical for accelerating post-fracture wound healing and aiding bone repair by facilitating the transport of osteoblasts and bone-forming factors through the vasculature (Zong et al., 2017). In bone marrow-derived mesenchymal stem cells (BMSCs), VEGF also increases the expression of bone morphogenetic protein 2 (BMP2) in endothelial cells, promoting osteogenic differentiation and mineralization via ALP. BMP-2 can further stimulate VEGF, potentially creating a positive feedback loop that encourages both vascularization and bone formation (Grosso et al., 2017). Copper (Cu) plays a pivotal role in modulating BMSCs and RUNX2, thereby promoting osteogenic differentiation by upregulating downstream genes associated with bone morphology (Liang et al., 2021). Optimal copper concentrations have been shown to inhibit osteoclast activity (Li and Yu, 2007; Bernhardt et al., 2021) and enhance osteogenic bioactivity by upregulating key components of the Wnt/β-catenin signaling pathway, including axin2, β-catenin, glycogen synthase kinase 3 (GSK-3β), LEF1, and TCF1/TCF7 (Tan et al., 2023; Vlashi et al., 2023). Previous studies have suggested that copper supplementation may inhibit the proliferation and differentiation of bone marrow stromal cells (BMSCs) (Li et al., 2014). It is hypothesized that this inhibitory effect is due to subtle variations in copper concentration, emphasizing a critical area for future research: the regulation of optimal copper concentrations within the broader context of bone metabolism.

The beneficial effects of optimal copper doses on bone metabolism are primarily attributed to their ability to form stable complexes with various drugs and compounds. This interaction not only enhances the therapeutic efficacy of these agents but also positively influences bone health, offering a valuable synergistic effect. Recent studies have highlighted an array of complexes and copper-related materials increasingly recognized for their significant roles in bone metabolism and overall bone health. Various compounds and materials, such as C/Cu complexes (Zhang et al., 2023), Silibinin-Cu(II)/Zn(II) (Vimalraj et al., 2018), Quercetin-Cu(II), Cu-mixed calcium phosphate cement (Lebedev et al., 2024), hydroxyapatite (Korowash et al., 2023; Noori et al., 2024), CS-BGNs (Wu et al., 2023a), peptide implant Cu coatings, Cu-Ti (Zhuang et al., 2021), CpTi-SiO2-3Cu (Ciliveri and Bandyopadhyay, 2024), Ti-5Cu (Zhao et al., 2022a), Cu-related hydrogels (Chen X. et al., 2024; Han C. et al., 2024; Zha et al., 2024), Zn/Mg/Cu-related metal-organic frameworks (MOFs) (Chen J. et al., 2024; Song et al., 2024), Cu2+/TA/HAP composite coatings (Haitao et al., 2024), Cu-MSNs (Hia et al., 2024a; Hia et al., 2024b), Cu-WH (Griesiute et al., 2024), and Cu-MBG (Anand et al., 2024), have demonstrated significantly greater efficacy compared to their single-use counterparts. Specifically, these materials enhance osteoblast activity, lysyl oxidase (LOX) activity, osteogenic differentiation of BMSCs, as well as angiogenesis and bacteriostasis within bone tissue. These effects collectively contribute to bone tissue repair, stabilization of bone metabolism, and improvement of bone strength. Copper-related complexes and materials show potential in treating OA; however, the comprehensive mechanisms regulating these treatments in bone and joint contexts remain unclear, and clinical evidence is still limited.

#### 2.2.3 Macrophage

Macrophages are key regulators in the pathogenesis and prevention of OA, with their interaction with inflammation being central to both the treatment and prevention of the disease (Chen et al., 2022). This discussion focuses on the critical role of copper (Cu) in modulating macrophage-mediated inflammation within the context of OA. Under physiological conditions, the balance between M1 and M2 macrophages is essential for maintaining metabolic homeostasis in the joint and synovial environment. In OA, the inflammatory and immune microenvironment disrupts this balance, leading to a significant increase in the M1/M2 macrophage ratio (Xu and Ji, 2023). M1 macrophages, as major contributors to OA-associated synovitis, produce reactive oxygen species (ROS), lactate dehydrogenase (LDH), reactive oxygen and nitrogen species (RONS) (Yan et al., 2020; Zhong et al., 2020), pro-inflammatory cytokines (Lin et al., 2019), nitric oxide (NO), and other harmful factors, while also upregulating matrix metalloproteinases (MMPs) (Ma et al., 2021). This activity not only intensifies inflammation and cartilage matrix degradation (Hu et al., 2019; Yan et al., 2021) but also amplifies oxidative stress-induced damage. The mutual reinforcement of M1 polarization and inflammation presents a significant challenge in OA treatment. Conversely, M2 macrophages exert effects that counteract those of M1 macrophages (Xu and Ji, 2023).

Numerous studies (Lin et al., 2019; Ma et al., 2021; Li S. et al., 2023) have demonstrated that optimal doses of copper can inhibit OA progression by modulating macrophage activity. The primary mechanism through which copper exerts its anti-inflammatory and protective effects in OA involves promoting the polarization and recruitment of macrophages from the pro-inflammatory M1 phenotype to the anti-inflammatory M2 phenotype (Li et al., 2023b). Copper-related materials, such as Cu-BSG (Lin et al., 2019), Cu-BGC (Lin et al., 2019), HPP@Cu gel (comprising sustained-release Cu, HA, PRP) (Zhu et al., 2022), CS-BGNs (Wu et al., 2023b), SA-Cu (Wu et al., 2024), Cu-Ti (Wang et al., 2019; Yunna et al., 2020; Chen L. et al., 2021), Cu-MOF nanomases (Yu et al., 2024), Cu-Zn BGNs (Huo et al., 2024), Ti-5Cu (Zhao et al., 2022b), 4-OI@Cu@Gel hydrogel (Zha et al., 2024), among others, including metal-organic frameworks (MOFs) (Li F. et al., 2022), have been shown to inhibit inflammation and oxidative damage while exhibiting antibacterial properties.These materials achieve these effects by promoting the proliferation and polarization of macrophages toward the M2 phenotype, thereby facilitating the repair of bone and cartilage and protecting osteoarthritic joints from further damage.

## 3 Abnormality of copper metabolism

The physiological role of copper in the body is tightly regulated, and both excess and deficiency of copper can lead to pathological conditions. Research shows that serum copper levels are generally elevated in patients with OA (Yang et al., 2023), suggesting a complex relationship between copper concentration and OA risk. Notably, copper deficiency has also been linked to a higher incidence of OA (Guan et al., 2023), underscoring the delicate balance required in copper homeostasis for joint health (Medeiros, 2016).

### 3.1 Copper deficiency

A follow-up study has suggested that copper deficiency might be an overlooked diagnostic factor, as it can significantly affect patient health by altering various metabolic indicators (Fujikawa and Haruta, 2023). Copper deficiency has widespread consequences on cellular and systemic metabolism due to its impact on the function of several essential enzymes, potentially leading to pathological conditions such as Menkes disease (Guthrie et al., 2020). Within the context of bone and cartilage, copper deficiency primarily results in detrimental effects due to the impaired function of copper-dependent enzymes (Sarazin et al., 2000), direct damage to bone and chondrocytes (Alshenibr et al., 2017; Liu et al., 2022), and decreased anti-inflammatoryand antioxidant defenses (Gaffney-Stomberg, 2019; Pantic et al., 2023).

Copper deficiency adversely affects the function of copper-dependent enzymes, including lysyl oxidase (LOX) (Vallet and Ricard-Blum, 2019), ceruloplasmin (CP) (Yiannikourides and Latunde-Dada, 2019), and superoxide dismutase (SOD) (Sun et al., 2024). The diminished activity of LOX severely disrupts collagen cross-linking, undermining the stability of the cartilage extracellular matrix and reducing bone strength (Sarazin et al., 2000; Alshenibr et al., 2017; Liu et al., 2022). Additionally, decreased SOD activity, a key enzyme in combating oxidative stress, results in a diminished capacity to clear reactive oxygen species (ROS) in bone and cartilage, leading to oxidative damage and subsequent deterioration of these tissues (Pantic et al., 2023).Furthermore, dietary copper deficiency exacerbates oxidative stress by reducing NRF2/HO-1 activity (Pan et al., 2024). From an osteogenic perspective, copper deficiency weakens bone integrity by depleting intracellular glutathione (GSH) (Bernhardt et al., 2021)and directly inhibiting osteoblast activity, thereby impairing bone remodeling (Pantic et al., 2023), while having minimal impact on osteoclasts (de Romaña et al., 2011; Tokuda et al., 2013). Collectively, these processes increase the susceptibility of bone and articular cartilage, heightening the risk of developing OA (Medeiros, 2016).

Moreover, copper deficiency influences iron metabolism by decreasing CP activity (Broderius et al., 2010; Yiannikourides and Latunde-Dada, 2019), leading to reduced extracellular iron excretion and intracellular iron overload (Miyajima, 2015; Zheng et al., 2018; Villadsen et al., 2023). This iron accumulation disrupts lipid metabolism (Ben-Hamouda et al., 2017; Raia et al., 2023) and heightens the risk of ferroptosis, a form of iron-induced cell death, particularly in chondrocytes. As ferroptosis contributes to OA development, exploring the interplay between copper deficiency, CP, iron metabolism, and cell death could offer a novel therapeutic approach to treating OA.

### 3.2 Cu overload

Research indicates that copper concentrations in both serum and joint fluid are markedly elevated in patients with OA compared to normal levels (Yazar et al., 2005; Yang et al., 2023), with degenerated cartilage showing higher copper content than healthy cartilage (Li et al., 2023c). These findings suggest a strong association between excessive copper and the development of OA, implicating it in cartilage damage (Guan et al., 2023; Li S. et al., 2023). *In vitro* studies of chondrocytes cultured with excess copper reveal abnormal cytoskeletal morphology, including altered microfilaments, microtubules, and fibers, as well as the inactivation of integrin-linked kinases (Zou et al., 2018), suggesting an aberrant state of chondrocytes (Terpstra et al., 2003). Elevated copper levels are also linked to the promotion of inflammatory states, which may exacerbate arthritis pathogenesis (Wang W. et al., 2023).Excess copper disrupts cellular function by competitively inhibiting the binding of cofactors to enzyme proteins, leading to redox imbalances (Guerra et al., 2023). In bone and cartilage, elevated copper levels damage osteoblasts, osteoclasts, and chondrocytes (León et al., 2014; Schamel et al., 2017; Bernhardt et al., 2021). Additionally, data have been compiled on the copper concentrations that induce damage to chondrocytes, osteoblasts, osteoclasts, macrophages, and bone marrow stromal cells (BMSCs) (Figure 1)

In osteoblasts, osteoclasts, and bone marrow stromal cells (BMSCs), excessive copper stabilizes the conformation of hypoxia-inducible factor 1-alpha (HIF-1α), leading to tissue hypoxia (Stelling et al., 2019). This condition downregulates the expression of Runt-related transcription factor 2 (Runx2) and decreases the levels of collagen type I, osteocalcin (OCN), insulin-like growth factor I (IGF-I), and bone morphogenetic protein 2 (BMP-2) (Qi et al., 2021). These molecular changes collectively impair the proliferative activity, osteogenic function, and osteogenic differentiation potential of osteoblasts. Additionally, excessive copper directly inhibits osteoclast function (Zofková et al., 2013; Bernhardt et al., 2021), thereby disrupting the balance between osteogenesis and osteoclast activity (Li et al., 2014). In chondrocytes, elevated copper levels suppress antioxidant enzymes, particularly superoxide dismutase (SOD) and glutathione peroxidase (GSH-Px), significantly reducing the cells’ antioxidant defense capacity. This suppression may facilitate the development of OA by promoting oxidative stress and related damage. Moreover, excessive copper induces a senescent phenotype in chondrocytes by impairing extracellular matrix synthesis (Massie et al., 1990).

Metallothioneins (MT-1 and MT-2), components of the metallothionein family, are stably expressed across various tissues. The expression of MT1/2 can be upregulated in response to stimuli such as copper excess, oxidative stress, and inflammation (Wake and Mercer, 1985), enabling the sequestration of excess copper ions for detoxification (Freedman and Peisach, 1989). The regulation of MTs may involve upstream factors such as hypoxia-inducible factor 2-alpha (HIF-2α), nicotinamide phosphoribosyltransferase (NAMPT), and the zinc transporter ZIP8-MTF1 axis (Park et al., 2023). This represents a cellular negative feedback mechanism against copper overload, establishing a critical link between copper and zinc metabolism (Park et al., 2023). Both metallothioneins (MTs) and glutathione (GSH) have significant potential in mitigating oxidative stress. Therefore, further exploration of copper metabolism and its related pathways may uncover potential therapeutic targets for treating related diseases, offering novel insights for clinical interventions.

### 3.3 Cuproptosis

Recent studies have identified a novel mode of programmed cell death induced by copper ions, referred to as “cuproptosis,” which may involve apoptosis, cysteine protease activity, or the accumulation of reactive oxygen species (ROS). This copper-mediated cell death, characterized by multiple factors and pathways, differs significantly from other forms of cell death, such as apoptosis and pyroptosis (Tsvetkov et al., 2022). Initially, excess copper binds to mitochondrial lipoic acid-related protein components (e.g., DLAT) (Rowland et al., 2018), leading to the formation of insoluble DLAT aggregates in mitochondria and the loss of FDX1-dependent Fe-S clusters. This process results in the oligomerization of DLAT and reduced Fe-S cluster stability, disrupting the tricarboxylic acid (TCA) cycle and triggering cell death through a protein toxicity-related stress response. Ferredoxin 1 (FDX1), identified as the central regulatory gene in copper-induced cell death, modulates this process by influencing the TCA cycle, specifically through the regulation of DLAT thioctyl acylation (Tsvetkov et al., 2022). Moreover, an excess of Cu^+^ ions can generate a substantial quantity of ROS through a mechanism similar to the Fenton reaction involving hydrogen peroxide (Ma et al., 2019), exacerbating oxidative stress-induced cellular damage. Elevated copper levels have been shown to activate the ROS/HO-1/NQO1 and NRF2/HO-1 pathways while concurrently inhibiting P38-MAPK-related signaling pathways (Fang et al., 2021a). ROS-induced damage to Fe-S cluster proteins, particularly the cyclical transformation between [4Fe-4S] and [2Fe-2S] clusters, is essential for maintaining equilibrium and adapting to changes in the redox environment (Read et al., 2021). However, substantial Cu^+^ reduction by histone H3/H4 in the nucleus further compromises Fe-S cluster proteins through ROS generation via a Fenton-like reaction.Proteins such as Ssq1, Jac1, and Nfs1 are integral to the assembly, maturation, and repair of mitochondrial Fe-S proteins. Copper-mediated superoxide dismutase (SOD) loss results in damage to these Fe-S cluster proteins (Strain et al., 1998). Additionally, copper can directly bind to glutathione (GSH), and excess copper leads to GSH depletion, a reduction in glutathione peroxidase 4 (GPX4) activity, and a subsequent decrease in cellular antioxidant capacity. Furthermore, excessive copper levels may enhance susceptibility to ferroptosis by affecting associated pathways, thereby exacerbating the progression of OA (Nong et al., 2023).In the subsequent sections, a comprehensive analysis will be conducted on how dysregulated copper metabolism influences ferroptosis sensitivity. Additionally, the interconnections between abnormal copper metabolism, cuproptosis, and ferroptosis will be elucidated.

**FIGURE 2:**
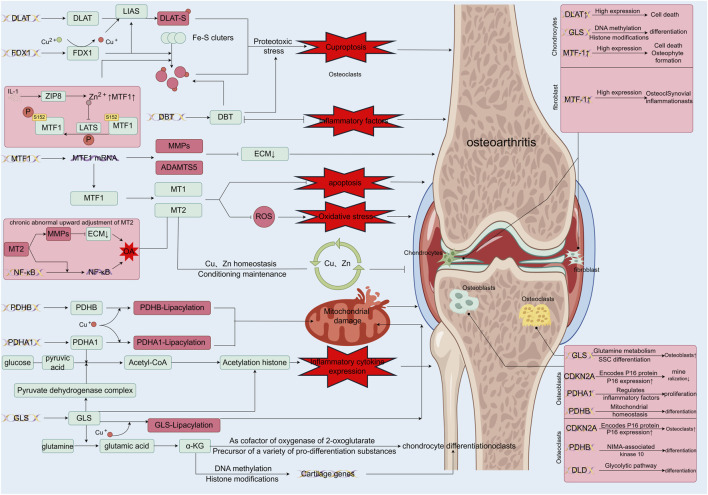
Functions of differentially expressed genes in copper-associated cell death and their role in osteoarthritis. FDX1 (Tsvetkov et al., 2022) initiates cytotoxic stress and induces copper death by reducing Cu^2+^, which is more toxic, and synergizes with DLAT mitochondrial proteins. This binding to lipidated proteins results in DLAT lipid acylation oligomerization, leading to the aggregation of lipidated proteins and the subsequent downregulation of iron-sulfur cluster proteins; MTF-1, a biomarker of OA, is regulated by Zn-related pathways, inflammatory factors, and mechanical stress. MTF-1 expresses MMP, ADAMTS5, and promotes catabolism, acting as an upstream regulator of MTs, affecting Cu and Zn homeostasis, and regulating oxidative stress; Under normal conditions, MTs form a network to sequester excess copper ions and act as a resistance factor to oxidative stress, but when MT2 is abnormally elevated, it promotes the expression of MMPs and activates the NF-κB pathway, leading to inflammatory injury (Park et al., 2023); PDHB and PDHA1, as key components of the pyruvate dehydrogenase complex, play pivotal roles in maintaining the tricarboxylic acid cycle in mitochondrial metabolism. In the presence of excess copper ions, the binding of copper leads to the accumulation of lipid-acylated proteins, resulting in mitochondrial metabolic dysfunction and subsequently inducing cell death (Rowland et al., 2018). Additionally, PDHB and PDHA1 catalyze the conversion of glucose-derived pyruvate to acetyl coenzyme A, thereby promoting the expression of inflammatory cytokines by regulating histone acetylation (Nie et al., 2022); GLS, another major component of the pyruvate dehydrogenase complex, disrupts mitochondrial metabolism upon binding to Cu (Tsvetkov et al., 2022). GLS regulates chondrogenic differentiation, ROS levels, and histone acetylation by modulating α-KG production, thereby influencing chondrogenic metabolism, cartilage differentiation, and ROS levels, leading to damage by affecting chondrocyte differentiation, among other factors (Yuan et al., 2022); DBT, one of the four enzymes lipoctanoylated by Cu, is situated in the enzyme complex at the TCA entrance and plays a vital role in maintaining mitochondrial TCA. Copper overload may lead to copper-induced cell death by affecting DBT (Wang X. et al., 2023), thereby promoting Fe-S disassembly and proteotoxic stress. Additionally, these related genes perform various regulatory roles in different cells of the bone and joint, such as chondrocytes, synovial macrophages, osteoblasts, and osteoclasts (Chen et al., 2023).

Recent genome-wide CRISPR/Cas9 screenings have identified seven genes upregulated in copper ion-induced cell death: ferredoxin 1 (*FDX1*), lipoyl transferase 1 (*LIPT1*), lipoic acid synthetase (*LIAS*), dihydrolipoic acid dehydrogenase (*DLD*), dihydrolipoic acid S-acetyltransferase (*DLAT*), pyruvate dehydrogenase E1 alpha subunit (*PDHA1*), and pyruvate dehydrogenase E1 beta subunit (*PDHB*). Simultaneously, three genes—metal-regulated transcription factor 1 (*MTF1*), glutaminase (*GLS*), and cyclin-dependent kinase inhibitor 2A (*CDKN2A*)—were downregulated. The regulatory interplay of these genes with copper metabolism offers insights into their roles in OA (Figure 2) (Chang et al., 2023).

## 4 Ferroptosis of chondrocytes in osteoarthritis

### 4.1 Ferroptosis

Fron-induced cell death, known as ferroptosis, is an iron-dependent mechanism characterized by the abnormal accumulation of lipid peroxides and their derivatives. Elevated levels of lipid peroxides, such as malondialdehyde (MDA) and the pro-inflammatory molecule 4-hydroxynonenal (4HNE), accumulate on cellular membranes and organelles, leading to membrane damage, rupture, and ultimately cell death (Chen X. et al., 2021). The Fenton reaction accelerates lipid peroxidation chain reactions, and under normal physiological conditions, these lipid peroxidation products are mitigated by the cell’s intrinsic reducing mechanisms, with selenium-dependent glutathione peroxidase 4 (GPX4) playing a critical role (Yang et al., 2020; Liu Y. et al., 2021). Glutathione (GSH) is a critical cofactor for GPX4 in this context. The reduction pathway also involves the SLC3A2 and SLC7A11 membrane transporters, essential for cystine uptake and subsequent GSH synthesis. Notably, Botulinum toxin A (Zeng et al., 2024) and Ruscogenin (Ruan et al., 2024), a bioactive compound from Radix Ophiopogon japonicus, have been shown to inhibit ferroptosis in chondrocytes via the SLC7A11/GPX4 pathway. While the GPX4 system is predominant, it is not the sole pathway; GPX4-independent mechanisms, such as the FSP1/CoQ10, DHODH, and GCH1 (GTP cyclohydrolase 1)/BH4 (tetrahydrobiopterin) pathways, also play significant roles (Bersuker et al., 2019; Gan, 2021; Yang H. et al., 2022). Moreover, elements like transferrin, the transferrin receptor, metal reductase 3, and FTH-1 are integral to the iron REDOX metabolic pathway (Park and Chung, 2019; Fang et al., 2021b; Yang M. et al., 2022; Ye et al., 2022). Mitochondrial dysfunction, due to its high iron content, is central to the pathogenesis of ferroptosis (Yang F. et al., 2022). This dysfunction exacerbates lipid peroxidation through mechanisms such as oxidative respiratory chain abnormalities, cysteine depletion, mitochondrial dihydroorotate dehydrogenase (DHODH) inhibition, and mitochondrial coenzyme Q10 (CoQ10) inhibition. As a prototypical ferroptosis inhibitor, nuclear factor erythroid 2-related factor 2 (*NRF2*) can upregulate various antioxidant enzymes, including heme oxygenase-1 (HO-1) and GPX4 (Lin Q. et al., 2023).

Ferroptosis is implicated in a wide range of physiological and pathological processes. The well-known tumor suppressor gene P53 can inhibit the transport of glutathione (GSH) precursors by downregulating the expression of SLC7A11 in the System Xc-transporter, thereby inhibiting GPX4 and inducing ferroptosis in cancer cells (Shao et al., 2022). Recent research has shown that P21 can stabilize GPX4 by modulating its recruitment to the linear ubiquitin chain assembly complex (LUBAC) and influencing the level of GPX4 M1-linked ubiquitination. Functionally, the knockdown of P21 enhances the sensitivity of chondrocytes to Erastin-induced ferroptosis, decreases Col2A1 expression, and increases MMP13 expression (Zheng et al., 2024). From an inflammatory perspective, both IL-1β and TNF-α have been shown to induce and enhance the expression of transferrin and its receptor, thereby increasing intracellular iron transport. Consequently, this inflammatory environment creates conditions that favor ferroptosis (Feng et al., 2020). This observation has led to investigations into the role of ferroptosis in the pathogenesis of OA. In the following sections, the relationship between ferroptosis and OA chondrocytes will be examined.

### 4.2 Ferroptosis of chondrocytes in osteoarthritis

A study examining the concentration of iron in articular fluid among patients with OA compared to healthy individuals revealed a significant elevation of iron levels in the articular fluid of those with OA (Yazar et al., 2005). Further research involving the knockout of the HFE gene, which regulates iron (Simão et al., 2019), and the observed increase in serum ferritin levels (Nugzar et al., 2018) has also established a link between iron dysregulation and OA. Moreover, prior research has demonstrated that chondrocyte ferroptosis contributes to the progression of OA, as evidenced by both *in vivo* and *in vitro* experiments. This phenomenon is not solely due to exposure to ferric ammonium citrate (FAC), which induces iron overload and subsequent ferroptosis. Notably, chondrocyte ferroptosis has been observed in a prototypical IL-1β-induced inflammation model of primary mouse chondrocytes. In this model, expression levels of type II collagen, System Xc, and GPX4 protein decreased, while expression of MMP13, ACSL4, and P53 increased (Yao et al., 2021). Recent *in vitro* experiments have shown that gossypol acetic acid (GAA) induces resistance to ferroptosis in chondrocytes within OA rat models by inhibiting GPX4 methylation (Shang et al., 2024b).

Fer-1, a lipophilic antioxidant, has been effective in mitigating Erastin-induced ferroptosis by clearing lipid peroxides (Dixon et al., 2012). Conversely, research indicates that desferrioxamine (DFO), an iron chelator, inhibits the development of IL-1β-induced chondrocyte models and slows disease progression in OA mouse models (Guo et al., 2022). DFO has been shown to inhibit ferroptosis through iron chelation, similar to the mechanism of Ferrostatin-1 (Fer-1) in mitigating lipid peroxidation. Additionally, both DFO and Fer-1 were found to enhance the expression of GPX4, HO-1, and other antioxidant enzymes by promoting the nuclear translocation of NRF2 in chondrocytes. This action reduces chondrocyte injury in OA mouse models, increases type II collagen expression, and decreases MMP13 levels (Guo et al., 2022). NF-κB, a key regulator of inflammatory processes, plays a significant role in OA pathogenesis by mechanisms involving both inflammation and ferroptosis. Upon activation, NF-κB induces the expression of HIF-2α and exacerbates ferroptotic activity by downregulating glutathione (GSH) levels and directly inhibiting the activity of GPX4. Furthermore, D-mannose has been shown to protect chondrocytes against ferroptosis by downregulating HIF-2α and promoting GPX4 expression (Zhou et al., 2021; 2022).

The transient receptor potential vanilloid 1 (TRPV1) has emerged as a promising therapeutic target in OA management. TRPV1 activation has been found to inhibit the ferroptosis pathway in chondrocytes, thereby decelerating OA progression. In tandem with this, innovative therapeutic strategies have been developed, incorporating TRPV1-targeting nanomaterials, which have demonstrated substantial efficacy *in vivo* (Li et al., 2024; Lv et al., 2024). A clinical study identified heightened ferroptosis in chondrocytes located in weight-bearing regions of the joint, implicating mechanical stress as a key driver of OA pathology (Wang et al., 2022). Excessive mechanical stress initiates catabolic processes in chondrocytes and disrupts glutathione (GSH) synthesis *via*calcium influx through mechanically activated Piezo channels, ultimately impairing glutathione peroxidase 4 (GPX4) activity and promoting ferroptosis. Furthermore, *in vitro* studies using IL-1β-induced chondrocyte models revealed that moderate mechanical stress activates the NRF2/GPX4/HO-1 axis while inhibiting the NF-κB P65 pathway, thus alleviating chondrocyte inflammation and ferroptosis (Han J. et al., 2024b). This evidence underscores the dualistic effect of mechanical stress on chondrocyte ferroptosis and provides a promising foundation for future research and therapeutic advancements.

Transferrin receptor 1 (TFR1), a transmembrane glycoprotein widely expressed across various tissue cells, plays a pivotal role in binding and facilitating the cellular uptake of ferric ions, which are subsequently reduced to ferrous ions by metal reductase 3. Recent studies have demonstrated that modulating TFR1 expression can influence ferroptosis inOA chondrocytes by regulating iron homeostasis. In a murine model of OA established through DMM surgery, researchers observed a significant increase in the positive rate of TFR1 expression within the model group (Wang W. et al., 2024). Further *in vivo* experiments using IL-1β-induced primary chondrocytes from mice revealed a dose-dependent increase in TFR1 expression, establishing a positive correlation between TFR1 expression and OA progression.Knockout of the TFR1 gene led to reduced reactive oxygen species (ROS) levels, increased expression of ferroptosis markers GPX4 and SLC7A11 (Wang J. et al., 2024), decreased expression of cartilage catabolic markers MMP3 and MMP13, and upregulation of anabolic markers COL2A1 and SOX9. Mitochondrial dysfunction, which plays a central role in iron metabolism, can trigger both inflammatory changes and ferroptosis in chondrocytes. Mitochondrial damage, along with the release of mitochondrial DNA (mtDNA) into the cytoplasm, can activate inflammatory responses via the cGAS-STING signaling pathway (Dela Cruz and Kang, 2018). The knockout of TFR1 has been shown to restore mitochondrial morphology by inhibiting IL-1β-induced cGAS/STING activity and mitigating mtDNA/cGAS/STING-mediated mitochondrial damage caused by iron overload (Wang W. et al., 2024). These findings establish TFR1 as a critical target for therapeutic intervention in ferroptosis related to OA and enhance the understanding of chondrocyte behavior in OA through the perspective of iron metabolic pathways. Such insights provide valuable directions for future research and clinical strategies.Recent studies have demonstrated that the overexpression of FTH-1, both *in vitro* and *in vivo*, enhances chondrocyte resistance to ferroptosis and inhibits cartilage extracellular matrix degradation. This is achieved through the regulation and inhibition of the MAPK pathway in the reversal of the DMM model, as well as the upregulation of SOX9 and Acan. Consequently, these mechanisms collectively contribute to a therapeutic effect on osteoarthritis (Yuan et al., 2024). A study investigating the role of GPR30 in ferroptosis within osteoarthritic (OA) cartilage demonstrates that the activation of GPR30 enhances the resistance of OA chondrocytes to ferroptosis. This effect is mediated through the modulation of YAP1 phosphorylation and the upregulation of FTH-1 expression, as validated under controlled conditions using G1 agonists and G15 inhibitors (Zhao et al., 2024).

Research has demonstrated that elevated levels of free fatty acids are present in patients with OA before the onset of classical symptoms (Bonner et al., 1975). The accumulation of lipid droplets in the cartilage matrix and chondrocytes accelerates cartilage degradation and the development of OA. Abnormal lipid metabolism within chondrocyte cytoplasm exacerbates cellular damage through various mechanisms (Zhou et al., 2021; Park et al., 2022). In the context of ferroptosis in chondrocytes, lipid metabolism is integral to the accumulation of lipid peroxides and subsequent oxidative stress, hallmarks of ferroptosis. Key enzymes such as ACSL4 and LPCAT3 play key roles in the synthesis of polyunsaturated fatty acid-containing phospholipids (PUFA-Pls), which are substrates for lipid peroxidation (LPO) (Klinger et al., 2002). ACSL4 facilitates the conjugation of long-chain polyunsaturated fatty acids (PUFAs), like adrenalic acid (ADA) and arachidonic acid (AA), with Coenzyme A (CoA) to produce ADA-CoA and AA-CoA. These conjugates are then re-esterified by LPCAT3 and incorporated into phospholipids (PLs), forming PUFA-PLs (Xu et al., 2023a). PKCβII enhances ACSL4 activity through phosphorylation, while acetyl-CoA carboxylase promotes PUFA synthesis. This process is driven by non-enzymatic autoxidation mediated by the iron-catalyzed Fenton reaction and enzymatic reactions involving ALOX or cytochrome P450 oxidoreductase, which further propagate lipid peroxidation (LPO). The combined effects of these processes lead to LPO, ultimately resulting in ferroptosis of chondrocytes in OA.

## 5 Disrupted copper homeostasis and ferroptosis

**FIGURE 3:**
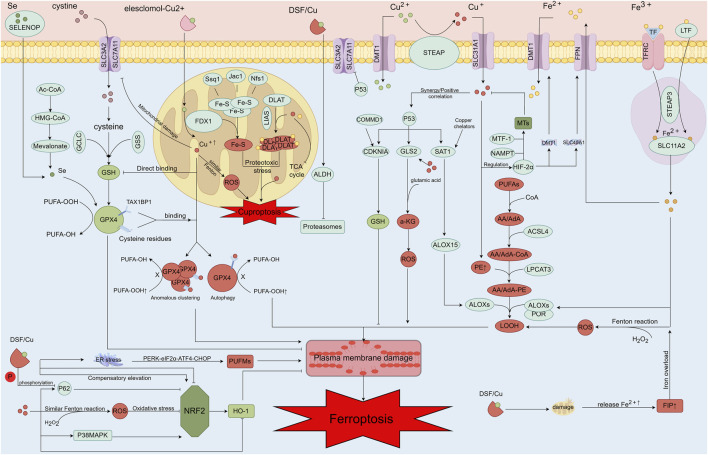
Mechanisms of copper ion-associated cell injury, including copper-induced ferroptosis and increased sensitivity to ferroptosis. After entering the cell via Elesclomol, copper ions directly target FDX1, converting Cu^2+^ into the more toxic Cu^+^. This conversion affects the oligomerization of DLAT and the disassembly of Fe-S clusters, leading to the copper-induced cell death process. Additionally, copper can directly bind to the cysteine residues of GSH, GPX4, and the TAX1BP1 receptor, resulting in the aberrant aggregation of GPX4 and the initiation of autophagy. This process inhibits the antioxidant activity of GPX4, thereby increasing the cell’s sensitivity to ferroptosis. A portion of copper can generate ferroptosis and enhance sensitivity to ferroptosis through the Fenton-like reaction, while another portion can induce ferroptosis through the Fenton reaction itself. Copper binding to GSH and GPX4 cysteine residues, as well as the TAX1BP1 receptor, leads to abnormal GPX4 aggregation and autophagy, further inhibiting GPX4’s antioxidant function and heightening the cell’s susceptibility to ferroptosis. Moreover, copper can produce a large amount of ROS through the Fenton-like reaction, damaging the mitochondrial oxidative respiratory chain and metabolism, and downregulating SLC7A11 expression. This downregulation leads to increased sensitivity to ferroptosis. Copper also decreases SELENOP levels, which in turn lowers selenium levels and reduces GPX4 activity. Under certain conditions, high concentrations of copper can interact with P53, exerting effects similar to those of P53. For example, copper can upregulate ALOX15 expression via SAT1, promoting lipid peroxidation and ferroptosis. P53 further promotes ferroptosis by regulating α-KG production through GLS2, increasing ROS production, and directly inhibiting SLC7A11. Copper can also stabilize HIF-2α through metallothioneins (MTs) and GSH. MTF-1 and NAMP play regulatory roles in copper homeostasis through MTs. Copper regulates the expression levels of DMT1 and FPN genes via HIF-2α, thereby influencing ferroptosis sensitivity by affecting iron metabolism. Copper also impacts the antioxidant pathway NRF2/IRF2/IRFN through several mechanisms and modulates important proteins in the NRF2/HO-1 pathway. DSF/Cu may reduce cellular antioxidant capacity via this pathway, promoting ferroptosis. Mitochondrial damage caused by DSF/Cu results in the release of significant amounts of iron from mitochondria into the cytoplasm, increasing the free iron pool and potentially contributing to ferroptosis through cellular iron overload. The DSF/Cu damage mechanism also impacts the lipid peroxidation process by promoting ER stress through PERK-eIF2α-ATF4-CHOP pathway regulation and influencing PUFM content. High copper concentrations elevate PE content, and excessive PE promotes LOOH content, leading to lipid peroxidation.

Ferroptosis primarily arises from disruptions in the REDOX system, excessive lipid peroxidation, aberrant lipid metabolism, and abnormal iron overload, among other factors (Li K. et al., 2022; Vana et al., 2024). Dysregulated copper metabolism and the activity of copper-related proteins further impact key pathways and mechanisms involved in ferroptosis, increasing its susceptibility and occurrence. In the following sections, we will comprehensively analyze how abnormal copper metabolism promotes and regulates ferroptosis, along with the interactions between copper and iron metabolism and their implications for ferroptosis in OA. This discussion aims to provide insights into the mechanisms underlying OA and to inform the development of future therapeutic strategies for OA-related conditions (Figure 3).

### 5.1 SLC7A11/GSH/GPX4

The SLC7A11/GSH/GPX4 pathway is a vital antioxidant mechanism within cells, particularly in the context of ferroptosis (Jiang et al., 2021). Both ferroptosis and cuproptosis can impair this pathway through various mechanisms involving excess copper. Firstly, copper (Cu) can directly bind to cysteine residues on glutathione peroxidase 4 (GPX4), leading to abnormal aggregation of GPX4 (Xue et al., 2023a), loss of its antioxidant activity, and the initiation of GPX4 autophagy-related reactions via the TAX1BP1 receptor. This sequence ultimately results in autophagy-mediated ferroptosis (Xue et al., 2023b). Notably, knocking down autophagy-related genes ATG5 and TAX1BP1 partially upregulates GPX4 and mitigates Cu-induced ferroptosis (Xue et al., 2023a). Similarly, the administration of copper-chelating agents has been shown to reduce sensitivity to ferroptosis, highlighting copper’s role in this process.Copper can also directly bind to glutathione (GSH), the key cofactor for GPX4, thereby depleting GSH levels. This depletion significantly reduces GPX4 activity and enhances susceptibility to ferroptosis (Scheiber et al., 2013). GSH also plays a regulatory role in maintaining intracellular copper homeostasis by modulating ATP7A and ATP7B (Banci et al., 2010), and its depletion may further exacerbate intracellular copper accumulation. However, the inactivation of GPX4 in certain cells has been associated with enhanced antioxidant capacity (Viswanathan et al., 2017), potentially indicating a compensatory mechanism involving alternative antioxidant pathways. This compensatory response, although not yet fully understood, is expected to become a focal point of future research, with significant implications for strategies aimed at mitigating oxidative stress and ferroptosis.

Wilson’s disease, a genetic disorder of aberrant copper metabolism, leads to excessive copper accumulation in the liver and is associated with premature OA due to metabolic disturbances caused by high copper levels (Ye et al., 2017). In patients with Wilson’s disease, elevated copper levels are accompanied by increased selenium and selenium-related transport-associated glycoprotein (SELENOP) concentrations in hepatic cells, alongside reduced activity. This reduction is possibly due to excessive accumulation of APOE, which binds to *SELENOP* and inhibits its secretion (Jin et al., 2020). Research indicates an antagonistic relationship between copper (Cu) and selenium (Se) in inflammatory and physiological contexts (Baudry et al., 2020). Excess copper also inhibits selenium-related enzymes, such as GPX4 (Schwarz et al., 2020). Targeting selenium metabolism—either by increasing selenium content or enhancing SELENOP expression—may alleviate oxidative stress and ferroptosis induced by copper overload.Research into the interactions between copper (Cu), selenium (Se), and associated proteins is essential for understanding and addressing inflammatory and age-related diseases. MEMO1, which sequesters copper and binds iron (Dolgova et al., 2024), has been shown to enhance GPX4 activity when knocked down in cellular models (Dolgova et al., 2024), highlighting the complex interplay between copper and iron.

*SLC7A11* is a vital transporter for cystine uptake, essential for glutathione (GSH) synthesis and cellular antioxidant defense, thereby playing a key role in resisting ferroptosis. Excessive copper accumulation induces mitochondrial dysfunction, leading to increased reactive oxygen species (ROS) levels and DNA damage. This condition results in the direct degradation of *SLC7A11* (Lei et al., 2022), diminishing GSH synthesis and reducing glutathione peroxidase 4 (GPX4) activity, which ultimately triggers ferroptosis.

Conversely, copper deficiency also disrupts the SLC7A11/GSH/GPX4 pathway, increasing susceptibility to ferroptosis. Under such conditions, hypoxia-inducible factor 1-alpha (HIF-1α) has been shown to upregulate SLC7A11, a ferroptosis suppressor protein. However, when intracellular copper ion levels decrease, the stabilizing effect of copper on HIF-1α is reduced. This attenuation leads to the downregulation of *SLC7A11* and a subsequent reduction in GSH synthesis, thereby increasing cellular vulnerability to ferroptosis (Tan et al., 2021). Furthermore, COMMD10, a protein involved in copper metabolism, interacts with ATP7A, ATP7B to facilitate copper excretion, thereby down-regulating HIF-1α-associated *SLC7A11* and CP and enhancing sensitivity to ferroptosis (Yang H. et al., 2022).

### 5.2 FSP1-CoQ10

The FSP1 pathway offers an antioxidant defense mechanism distinct from the SLC7A11/GSH/GPX4 pathway. FSP1 works by interacting with DHODH to convert CoQ to CoQH2 via NAD(P)H. CoQH2 then neutralizes reactive oxygen species (ROS) at both the inner mitochondrial and plasma membranes (Koppula et al., 2022), thus reducing oxidative stress and preventing ferroptosis. Excess copper exposure can inhibit FSP1 expression, leading to decreased CoQ levels. This reduction impairs cellular antioxidant defenses and the clearance of ROS, thus contributing to ferroptosis (Zhong et al., 2024). This disruption in the FSP1-CoQ10 pathway due to excessive copper introduces a novel mechanism of copper-induced ferroptosis, distinct from the traditional SLC7A11/GSH/GPX4 pathway, and presents a potential target for therapeutic interventions aimed at modulating ferroptosis.

Additionally, excessive copper has been shown to elevate the protein expression levels of ACLS4 and NCOA4 (Zhong et al., 2024). ACLS4 promotes ferroptosis through fatty acid metabolism (Liu Z. et al., 2021; Zhao et al., 2021), while NCOA4 facilitates ferroptosis through ferritinophagy (Li et al., 2020). Moreover, NRF2 is crucial in regulating the expression of ferroptosis-related genes (Dodson et al., 2019; Li et al., 2020) and mitigating oxidative stress. Excess copper may downregulate *NRF2* expression by promoting its inhibitor, Keap1, potentially contributing to ferroptosis (Zhong et al., 2024). Further research is needed to validate these mechanisms in osteoarthritic chondrocytes and explore their implications for therapeutic strategies.

### 5.3 ATP7A, ATP7B, CP

The imbalance between ATP7A/B, which are essential for copper excretion and cellular endocrine functions (Dmitriev and Patry, 2024), and CTR1, the main copper uptake transporter (Ross et al., 2024), can significantly impact copper and iron metabolism, thereby influencing ferroptosis sensitivity (Li et al., 2023b). Elesclomol, a small molecule that binds Cu^2+^, targets and degrades ATP7A. This degradation leads to abnormal copper accumulation within the cell, which promotes the production of reactive oxygen species (ROS) through the Fenton reaction. The resulting oxidative stress damages mitochondria and degrades SLC7A11, diminishing GPX4 activity and ultimately triggering ferroptosis (Halliwell and Gutteridge, 1984; Kirshner et al., 2008; Gao et al., 2021).

Ceruloplasmin (CP) plays a pivotal role in copper transport and iron homeostasis by oxidizing Fe^2+^ to Fe^3+^, which facilitates iron binding to transferrin and enhances intracellular iron excretion (Vashchenko and MacGillivray, 2013). CP also helps mitigate iron overload and ROS production via the Fe^2+^-mediated Fenton reaction (Bakhautdin et al., 2014). In OA, severe oxidative stress can reduce CP levels (Kvien, 2010). This reduction can lead to iron overload, increasing the risk and sensitivity to ferroptosis (Mo et al., 2024). Additionally, copper deficiency can reduce CP levels, potentially disrupting iron metabolism and contributing to OA susceptibility (Rolić et al., 2024). Further research is needed to validate these mechanisms in the context of OA and explore potential therapeutic interventions.

### 5.4 P53/SAT1/GLS2

Research suggests that P53 plays a pivotal role in regulating copper homeostasis (Xiong et al., 2023) and heightens sensitivity to copper-induced cell death by inhibiting glycolysis (Liu et al., 2019; Tsvetkov et al., 2022). Elevated copper levels activate P53, which subsequently downregulates SLC7A11 (Huang et al., 2023) and upregulates SAT1 and GLS2, ultimately leading to ferroptosis. SAT1 further amplifies the expression of arachidonic acid 15-lipoxygenase (ALOX15), intensifying oxidative stress and lipid peroxidation, thereby increasing cellular vulnerability to ferroptosis (Ou et al., 2016). GLS2, in concert with P53, promotes the conversion of glutamate to α-ketoglutarate (α-KG) and induces the production of reactive oxygen species (ROS). In hepatic cells lacking STP7B, GLS2 levels significantly rise under elevated copper conditions, suggesting that high copper concentrations may exert effects on GLS2 similar to those mediated by P53 (Yin et al., 2022). Thus, P53 may act as a key intermediary linking dysregulated copper/iron metabolism to ferroptosis, making it a potential target in conditions associated with copper overload and resistance to ferroptosis. Moreover, excessive copper directly damages mitochondrial DNA, causing extensive mitochondrial damage. This mitochondrial impairment leads to the release of free iron and the formation of multiple free iron pools, thereby greatly increasing susceptibility to ferroptosis (Ren et al., 2021).

## 6 Discussion

Copper and iron homeostasis are essential for maintaining normal physiological functions. In the context of bone and cartilage health, copper homeostasis plays a critical role in protecting chondrocytes, regulating osteoblast balance, modulating M1 and M2 macrophage polarization, and promoting the differentiation of bone marrow-derived mesenchymal stem cells (BMSCs). This paper provides a comprehensive review of the protective effects of optimal copper doses on bone and cartilage damage, along with an analysis of the detrimental impacts of abnormal copper metabolism, including copper overload and deficiency. The findings indicate that even slight variations in copper levels significantly affect bone and cartilage metabolism. Moreover, an exploration of the interplay between copper and iron metabolism, as well as the mechanisms of copper- and iron-induced cell death, reveals a close and potentially synergistic relationship between these metabolic pathways.Whether through a reduction in the activity of antioxidant copper enzymes, such as ceruloplasmin (CP) and superoxide dismutase (SOD), due to copper deficiency, or the generation of reactive oxygen species (ROS) via Fenton-like reactions triggered by copper overload, the outcomes are consistent: abnormal regulation of anti-ferroptosis factors and increased cellular susceptibility to ferroptosis due to mitochondrial damage. Consequently, the initiation and progression of cellular ferroptosis may contribute to increased susceptibility to OA and the advancement of the disease. Elevated copper levels in blood and synovial fluid have been associated with the pathogenesis of OA, and chondrocyte ferroptosis has been identified as a potential therapeutic target. Therefore, it is hypothesized that elevated copper concentrations may damage bone and cartilage cells via ferroptosis, thereby accelerating OA progression.Furthermore, copper deficiency might increase the risk of OA by enhancing ferroptosis vulnerability through decreased CP and antioxidant kinase activity. The SLC7A11/GSH/GPX4 pathway, a common mechanism underlying both copper- and iron-induced cell death resulting from abnormal copper metabolism, is of significant relevance to Cu/Fe-related injury. This pathway warrants further investigation for its potential implications in the treatment and prevention of OA. Additionally, regulating copper-related proteins, such as CP , ATP7A and ATP7B, is essential for maintaining copper and iron balance, as well as for the prevention, treatment, and repair of OA-related injuries.
